# Herbivore cues and plant damage-associated compounds jointly alter seed germination and seedling herbivory

**DOI:** 10.1007/s00442-025-05831-z

**Published:** 2025-11-25

**Authors:** Katherine M. Overstrum, Eirette M. Santiago, Brooke A. Pellegrini, Kevin C. Headrick, Colin M. Orians, John L. Orrock, Evan L. Preisser

**Affiliations:** 1 Department of Biological Sciences, University of Rhode Island, Kingston, RI 02881 USA; 2 Department of Biology, Tufts University, Medford, MA USA; 3 Department of Integrative Biology, University of Wisconsin, Madison, WI USA

**Keywords:** Kairomone, Signal, Mollusk, Risk, Induced defense

## Abstract

While plant defense against herbivory is primarily thought to occur following attack, there is also evidence that plants can detect and respond to pre-attack cues. These cues include chemicals released from damaged conspecifics and kairomones, non-attack-related substances emitted by an herbivore that plants can detect and use to their benefit. It is unknown, however, whether or how plants react to the interaction of these pre-attack cues. We measured germination, growth, and herbivore susceptibility of *B. nigra* seedlings in an experiment that crossed the presence/absence of crushed *B. nigra* leaves with the presence/absence of mucus of a generalist herbivore, *A. subfuscus*. Seeds exposed to both crushed leaves and slug mucus germinated 8% more quickly than control seeds; neither risk cue increased germination speed when tested individually. The same pattern was found in herbivore bioassays: *Spodoptera exigua* ate almost 10 × more foliage from control seedlings than from seedlings exposed to both crushed leaves and slug mucus. There was no difference in the final biomass of mature plants, suggesting that plants exposed to herbivore cues early in their development can increase defense without a measurable cost in size at maturity.

**Supplementary Information:**

The online version contains supplementary material available at 10.1007/s00442-025-05831-z.

## Introduction

Plants threatened by herbivores must balance the benefits of defense with its energetic costs. The modular design of plants, paired with the lack of a central vascular system and the highly plastic nature of meristem tissue, means that low levels of herbivory pose little risk to survival and fitness (Karban & Agrawal 2002). As a result, mature plants can generally afford to wait until attack begins to induce chemical and/or structural defenses to prevent further damage (Karban 2020). This risk-tolerant approach is less suitable for juvenile plants such as seeds and seedlings, that either lack the energetic reserves to recover from damage or are small enough to be eaten in their entirety before they have the chance to respond. As a result, the ability to detect and respond to pre-attack herbivore cues may be critically important to young plants. Since allocation to defense can reduce plant growth and lead to young plants losing out in competition for light and other resources, selection should favor individuals capable of balancing these competing demands and determining whether a given stimulus warrants the induction of pre-attack defense (Karban & Orrock 2018).

Plants seeking to detect pre-attack herbivore cues may take advantage of kairomones, non-attack-related chemical cues incidentally emitted by consumers that are detected by and benefit prey. Kairomones include both specific chemicals and mixtures and are often byproducts associated with daily life such as excrement, sex attractants, and fluids associated with locomotion or oviposition. Exposing *Brassica nigra* seeds and seedlings to snail (*Helix aspersa*) locomotion mucus, for example, decreased plant growth and herbivory relative to control plants (Orrock 2013); subsequent work found that the effect of *H. aspersa* mucus on tomato seedling growth and defense rivaled that of the plant defense elicitor methyl jasmonate (MeJA) (Orrock et al. 2018). Exposing *Solidago altissima* to sex attractants of the galling fly *Eurosta solidaginis* reduced herbivory and subsequent oviposition in both laboratory and field experiments (Helms et al. 2013). Insect frass from multiple herbivores has similarly been shown to induce defense in several host plant species (Ray et al. 2016).

Plants may also assess the risk of herbivore attack via ‘damaged self’ cues produced by nearby injured conspecific or heterospecific individuals. These damage-associated molecular patterns (DAMPs) are chemicals released by stressed or injured cells that alert nearby cells to a potential threat. Pattern recognition receptors (PRRs) located on the cell surface and spanning the plasma membrane detect damage and respond via increased production of reactive oxygen species, protein kinases, and hormones such as jasmonic acid (JA), salicylic acid (SA), and ethylene (Tanaka & Heil 2021). Since PRRs are most responsive to species-specific cues, defensive induction is greatest in response to DAMPs from the same or closely related species (Duran-Flores & Heil 2018). Bean (*Phaseolus vulgaris*) plants, for instance, increased reactive oxygen species production and JA induction in response to leaf homogenates from conspecific—but not heterogeneric—plants (Duran-Flores & Heil 2014). Although a number of chemical compounds that act as DAMPs have been identified (Tanaka & Heil 2021), DAMPs detected by individuals growing nearby a wounded plant would be accompanied by a mixture of other compounds found in damaged plant material.

Although plant responses to non-damage herbivore kairomones and DAMPs have received considerable attention, the interaction between the two has only been explored in animal predator–prey systems. Studies of the separate and combined effects of both kairomones and alarm cues in several tadpole species found that while exposure to either alarm cues or kairomones induced prey defense, their combination elicited the most complete and effective set of antipredator responses (Schoeppner & Relyea 2005; Schoeppner & Relyea 2009a, b). Prey responded more strongly to a kairomone–DAMP mix derived from predators fed conspecifics than those fed distantly related species, suggesting that the phylogenetic similarity of predator-consumed individuals is also important (Schoeppner & Relyea 2005). Similar results have been found in work exploring antipredator responses in the aquatic macroinvertebrate *Caecidotea intermedius* (Spivey et al. 2015) and *Daphnia magna* (Pestana et al. 2013; Ślusarczyk 1999; Wieski & Slusarczyk 2022), suggesting that kairomones and DAMPs together generally provide a reliable indication of predation risk.

We describe the results of work examining how kairomones, DAMPs, and their interaction affect seed germination and seedling growth, chemistry, and herbivore palatability. Seeds and seedlings of our study species, *Brassica nigra*, have been shown to induce defense in response to locomotion mucus from the generalist molluscan herbivores *Helix aspersa* (Orrock 2013) and *Arion subfuscus* (Pellegrini et al. 2024). Our study crossed a kairomone treatment (control or locomotion mucus from *A. subfuscus* fed *Brassica nigra*) with DAMP (*B. nigra* leaf homogenate) presence/absence and measured various aspects of plant response. The results demonstrate that plants can utilize both signals and reveal an ecologically relevant story of growth and defense under exposure to pre-attack cues.

## Materials and methods

### Study species

*Arion subfuscus* is native to Europe and was introduced to North America in the early 1900s (Chichester & Getz 1973). It is an important molluscan herbivore and in the invaded range (Chichester & Getz 1969) and the most abundant species of slug in New England (French 2012). Adults were collected May–July 2024 from forested areas in South Kingstown, RI, USA. Slugs were maintained in six large soil- and detritus-filled terraria at 21–24 °C on a diet of organic lettuces, carrots, and field-collected seedlings of various plant species. The terraria were misted daily in order to maintain high humidity levels, and decaying plant material was removed when necessary.

*Brassica nigra* is a fast-growing plant native to the Mediterranean that is naturalized worldwide and grows wild in RI. Seeds were sourced from Outsidepride Seed Source, LLC (Independence, OR, USA).

*Spodoptera exigua* is a well-known pest with a broad host range. Larvae typically feed on foliage and fruit, and have been used in many studies because of their generalist diet, consistent feeding behavior, and quick life cycle (Merkx-Jacques et al. 2008). *S. exigua* used in this experiment were sourced as eggs from Frontier Agricultural Services (Newark, DE, USA) and reared in deli cups and maintained on an artificial diet until choice studies commenced.

### Experimental design

The 2 × 2 experimental design crossed two herbivore-risk treatments (Kairomone) with two damage-associated molecular pattern (DAMP) treatments for a total of four treatments. The herbivore-risk treatments were mucus from *A. subfuscus* fed *B. nigra* for 3 days (+Kairomone) or a no-mucus control (-Kairomone). The two DAMP treatments were water mixed with ground *B. nigra* leaves (+DAMP) or water alone (-DAMP). Each treatment was applied to *B. nigra* seeds and plants and their responses were measured.

### Herbivore-risk treatment

Three days before preparing the risk treatments, our population of mature *A. subfuscus* was divided into multiple small plastic terraria lined with wet paper towels where they were fed lab-grown *B. nigra* seedlings. After 3 days, we flattened 4 g of a soil-and-distilled-water mixture (1:4 ratio) into each of multiple 90 mm petri dishes. In dishes assigned to either the high-risk or control treatments, a mature *A. subfuscus* was allowed to crawl on the soil for 24 h in a dark cabinet at 20–24 °C. Control plates were held in similar conditions, but did not receive a slug. All slugs were then returned to the large dirt-filled terraria and the petri dishes were frozen at -20 °C in a freezer for 2–4 weeks until they were defrosted and used. We used frozen rather than ‘fresh’ plates for the work because it would have been extremely difficult to generate the > 1000 ‘fresh’ risk treatment plates necessary for the work when the main experiment (detailed below) was running. To ensure that previously frozen slug mucus induced similar plant responses as freshly deposited mucus, we conducted a pilot experiment confirming that mucus frozen for up to 6 months accelerated *B. nigra* seed germination (Online Resource 1).

### DAMP treatment

*Brassica nigra* seeds were sown in potting soil and grown in trays on a sunny windowsill. To prepare the leaf homogenate for the DAMP+ treatment, *B. nigra* seedlings (~ 3 cm in height) were cut at the base of the stem and blended with distilled water (1:30 ratio by weight, as per Duran-Flores and Heil 2014) in a blender (Waring Commercial, Torrington, CT, USA) and then allowed to sit for 2 h before use as per Duran-Flores and Heil (2014). A water-only control solution was also prepared for use in the -DAMP treatment.

#### Seed responses

##### Germination assay

On 15 July, 16 petri dishes from each of the two herbivore-risk treatments were removed from the freezer. Once fully defrosted, a piece of 90 mm white filter paper was placed on top of the soil inside each of the dishes. Half of the dishes in each herbivore-risk treatment were then wetted with either 1 mL of the leaf homogenate solution (+ DAMP) or 1 mL of distilled water (-DAMP). Each of the four treatments was thus replicated eight times for a total of 32 dishes.

Immediately after the addition of the DAMP solution, 50 *B. nigra* seeds were placed on top of the filter paper in each dish. Dishes from all four treatments were then interspersed and placed in a dark cabinet at 21 °C. Starting 15 h after seed placement and continuing every 3 h afterwards, the seeds in each petri dish were checked for germination. We chose to start germination checks at 15 h on the basis of prior work that found virtually no seed germination before this point (Pellegrini et al. 2024). At each interval, each seed was carefully checked for radicle emergence. Seeds with a radicle were counted and then removed from their petri dish. The checks continued until hour 45, when at least 90% of seeds from each treatment group had germinated.

#### Seedling responses

##### Planting, growth, and cue application

Prior to the start of the experiment, 320 6 × 6 cm black plastic pots were filled with Miracle-Gro potting mix (Marysville, OH, USA). The pots were placed in 14 square boxes (36 cm × 36 cm) with a mesh bottom. Each box contained six plants from each treatment, for a total of 36 pots per box in 13 of the 14 boxes; the 14th box had empty pots added to the ‘empty’ slots in it.

The first 80 seeds to germinate in each of the four treatments (320 total pots) were removed and buried individually 1 cm under the soil in a pot that was then placed in a box. The boxes were placed along a windowsill where they received approximately 7 h of direct sunlight per day. Each box was rotated clockwise every other day, and every week each pot within each box was rotated within its box by moving it one slot down and one slot to the right. Each plant received 5 mL of water daily, administered via pipette at the base of the plant.

Starting 1 week after germination, one petri dish of soil from the appropriate herbivore-risk treatment was removed from the freezer, defrosted, and the soil within it added carefully to the base of a plant from the appropriate treatment. Immediately after the soil had been applied, each plant was watered with 5 mL of freshly prepared leaf homogenate (+ DAMP) or control (-DAMP) solution. Seedlings were subsequently watered every 2 days with 5 mL of water. The herbivore-risk and DAMP treatments were applied weekly for a total of 5 weeks; each plant received the same cue combination for the duration of the experiment.

### Biomass and glucosinolate analysis

On day 36 of the experiment (i.e., one day following the final cue application), 20 plants from each treatment (80 total plants) were destructively harvested. We first cut the third-newest leaf from each plant at the petiole, weighed it, then rolled it into a 15 mL falcon tube that was capped, flash frozen using liquid nitrogen, and placed in a −80 °C freezer for later glucosinolate analysis.

The rest of the plant was removed by hand from the soil and cut at the cotyledon scar. The aboveground material was immediately placed in a coin envelope; the below-ground material was carefully rinsed in water and patted dry before doing so. All samples were then placed in a drying oven (Blue M Electric Company, Blue Island, IL, USA) for 4 days at 60 °C before weighing the above- and below-ground portions of each plant. A separate set of leaves was cut from unused plants and weighed immediately before being dried and re-weighed; we used the resulting wet:dry weight regression to convert the wet weight of the leaf removed for glucosinolate analyses into dry weight for addition to the aboveground biomass.

Glucosinolates were extracted and analyzed using standard methods (Pieck et al. 2015; Brown et al. 2003). Briefly, frozen samples were ground under liquid nitrogen in a Retsch MM400 ball-mill grinder (Retsch GmbH, Haan, Germany). Once ground, 70–120 mg of frozen leaf tissue in 2 mL microcentrifuge tubes was extracted in 1 mL methanol. Glucosinolates were bound to 20 mg of a DEAE Sephadex A25 pellet. Sulfatase (4 mg/mL) was then used to cleave desulfoglucosinolates from the pellet, and the supernatant was filtered, 0.22 µm, into HPLC vials. A sinigrin standard was processed using the same method; 90% of the glucosinolates in *Brassica nigra* are sinigrin (Chaplin-Kramer et al. 2011; Feeny & Rosenberry 1982). The concentration of sinigrin was analyzed by HPLC using a 150 × 4.6 mm C18 reverse-phase column (Phenomenex, Torrance, CA) with a water acetonitrile gradient (Brown et al. 2003). Each sample was injected twice and the concentration of sinigrin (mg/g fresh leaf mass) was averaged for each sample.

### Spodoptera exigua bioassays

Five weeks after the start of the experiment, a series of paired-choice assays were performed in which final-instar *Spodoptera exigua* were allowed to choose between a detached leaf from a control (-Kairomone/-DAMP) plant and another leaf from one of the three remaining treatment groups (-Kairomone/+DAMP, +Kairomone/-DAMP, +Kairomone/+DAMP). We chose to use a detached leaf rather than a mature plant because foliage from the same plants was also being used for glucosinolate assays and biomass estimation. Since we were interested in herbivore preference (a short-term metric) rather than herbivore performance (a long-term metric), we did not weigh each final-instar larvae. *Spodoptera* were chosen as a specific alternative to *A. subfuscus* for these bioassays because they do not leave mucus behind on leaves after feeding, making damage-induced change to leaf weight easier to detect. There were 15 replicates for each of the three bioassay combinations (45 total bioassays).

For each paired-choice bioassay, a single *B. nigra* leaf from a control and one from a specific treatment plant was cut from a plant, weighed, and placed in a prepared petri dish for bioassays. Leaves of similar age and sizes were paired together whenever possible, and both plants were selected from the same box. For each bioassay, a 90 mm petri dish was lined with 90 mm white filter paper and sprayed twice with distilled water before the leaves were placed next to each other on opposite sides of the dish. One *S. exigua* was placed in the center of the dish, which was then closed and held in a dark cabinet for 24 h. At the end of the bioassays, both leaves were re-weighed. We also conducted no-herbivory bioassays (necessary to quantify ambient changes in leaf weight over time); these were identical to the choice assays except for the absence of *S. exigua* larvae.

### Statistical analysis

We initially examined the germination data using failure-time analyses (Cox proportional hazards model) but ultimately opted to use a generalized linear mixed model (GLMM) with a binomial response distribution because model diagnostics revealed that the proportional hazards assumption was violated. The model specified kairomone and DAMP treatments as fixed effects, the hour since the experiment was initiated as a covariate, and also included a cubic term for hour. We modeled time as non-linear because preliminary analyses indicated a non-linear relationship between probability of germination and the duration of the experiment and a cubic relationship was a significantly better fit than linear or quadratic based on comparison of AICc and log-likelihood tests. We explored all possible interaction terms. The combined herbivore bioassay dataset contained a few anomalously high and low datapoints spread across multiple treatments. Since these likely reflected errors in weighing/data entry, we removed the highest and lowest 2.5% of values from the combined dataset before analyzing each of the choice bioassays separately. For each choice bioassay, data on leaf proportion remaining per treatment were analyzed using GLM (identity link function) with treatment and bioassay date as fixed effects and plate identity nested within date as a random effect. We examined plant growth and glucosinolate concentration using linear models that specified kairomone and DAMP as fixed effects. Analyses were conducted in R version 3.6.1 (R Core Team 2019) and JMP version 18.1.2 (SAS Institute Inc. 2025).

## Results

### Seed germination

**Fig. 1:**
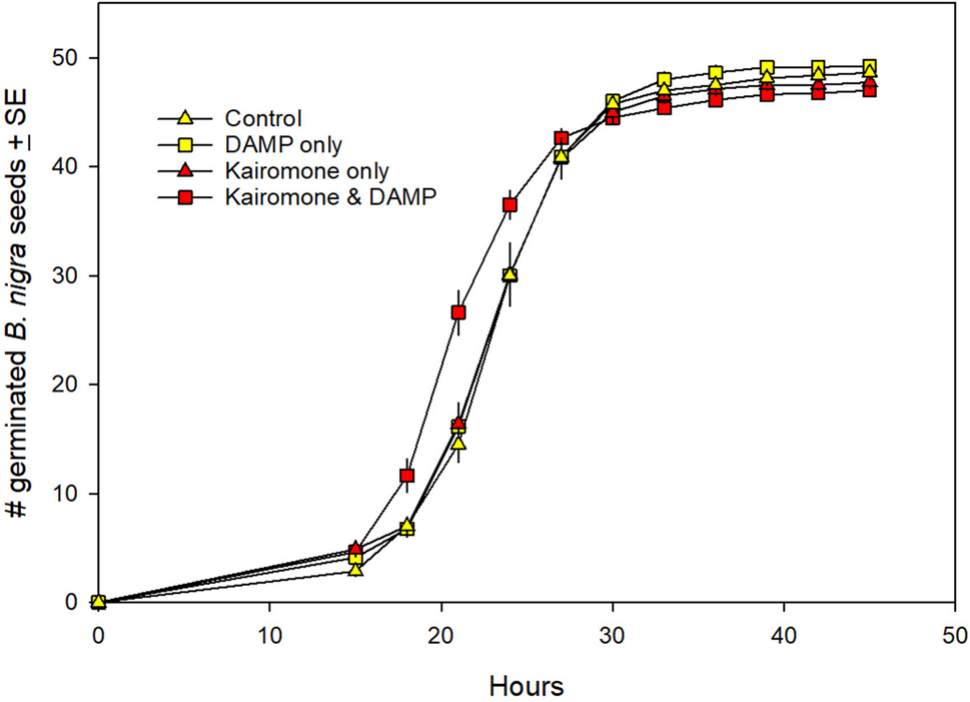
Effect of exposing *B. nigra* seeds to control (soil and water only), kairomone only, DAMP only, or both kairomone and DAMP prior to and during germination. Seeds exposed to both kairomone and DAMP germinated significantly faster

Patterns of germination in time were strongly cubic (X^2^ = 579.40, 3 d.f., *p* < 0.001) with patterns of germination accelerating early in the experiment and then leveling off as time proceeded (Online Resource 2). Germination was affected by kairomones and DAMPs, as reflected by a highly significant kairomone*DAMP*hour interaction (Fig. 1; X^2^ = 19.85, 3 d.f., *p* < 0.001), as well as a significant kairomone*hour interaction (X^2^ = 34.89, 3 d.f., *p* < 0.00) and a significant DAMP*hour interaction (X^2^ = 50.72, 3 d.f., *p* < 0.001). Averaged across these significant interactions with time, there was no main effect of DAMPs (X^2^ = 2.18, 1 d.f., *p* = 0.14), kairomones (X^2^ = 0.01, 1 d.f., *p* = 0.91) or the interaction between DAMPs and kairomones (X^2^ = 0.80, 1 d.f., *p* = 0.37). Seeds in the control treatment averaged 24.5 h to germination, while those in the kairomone-only treatment averaged 24.4 h (0.6% faster) and seeds in the DAMP-only treatment averaged 24.1 h (1.7% faster). In contrast, seeds exposed to both kairomones and DAMPs germinated in 22.6 h (7.9% faster than control seeds; Fig. 1).

### Seedling biomass

**Fig. 2:**
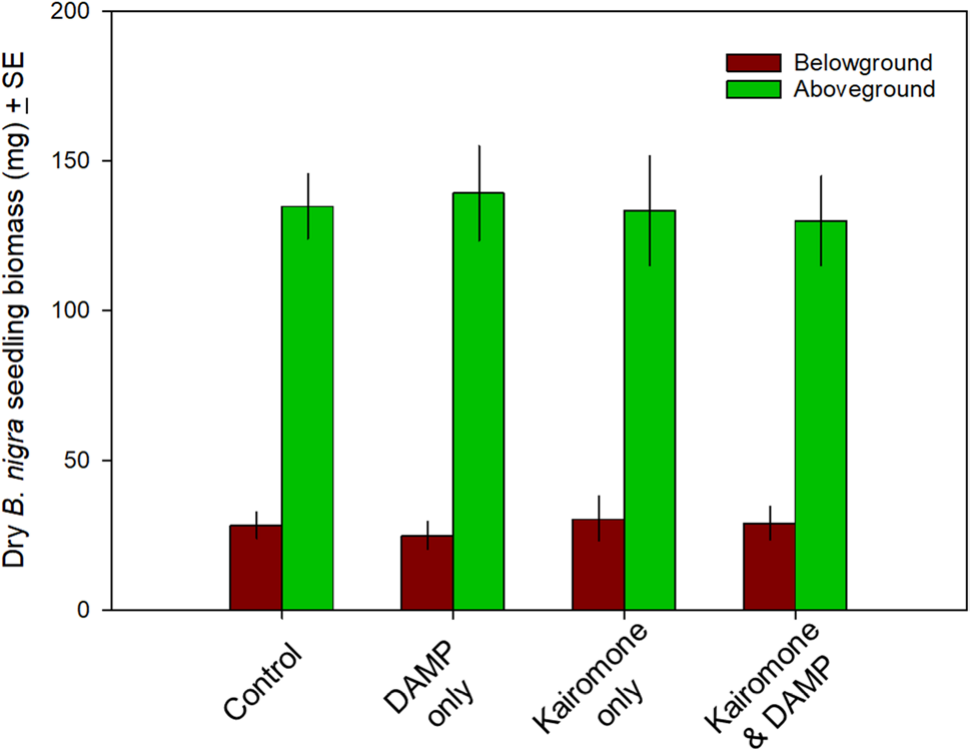
Effect of exposing *B. nigra* seeds and seedlings to control (soil and water only), kairomone only, DAMP only, or both kairomone and DAMP. There was no significant difference in aboveground, below-ground, or total biomass for seedlings at the time of harvest

Mean aboveground plant biomass (Fig. 2, green bars) was not affected by kairomones (F_1,82_ = 0.013, *p* = 0.72), DAMPs (F_1,82_ = 0.04, *p* = 0.97), or their interaction (F_1,82_ = 0.07, *p* = 0.80). Mean below-ground plant biomass (Fig. 2, brown bars) was similarly unaffected by kairomones (F_1,81_ = 0.30, *p* = 0.59), DAMPs (F_1,81_ = 0.18, *p* = 0.67), or their interaction (F_1,81_ = 0.03, *p* = 0.86).

### Glucosinolate concentrations

There was no effect of kairomones (F_1,76_ = 1.62, *p* = 0.21), DAMPs (F_1,76_ = 0.004, *p* = 0.99), or their interaction (F_1,76_ = 1.06, *p* = 0.31) on leaf glucosinolate concentration.

### Herbivory bioassays

**Fig. 3:**
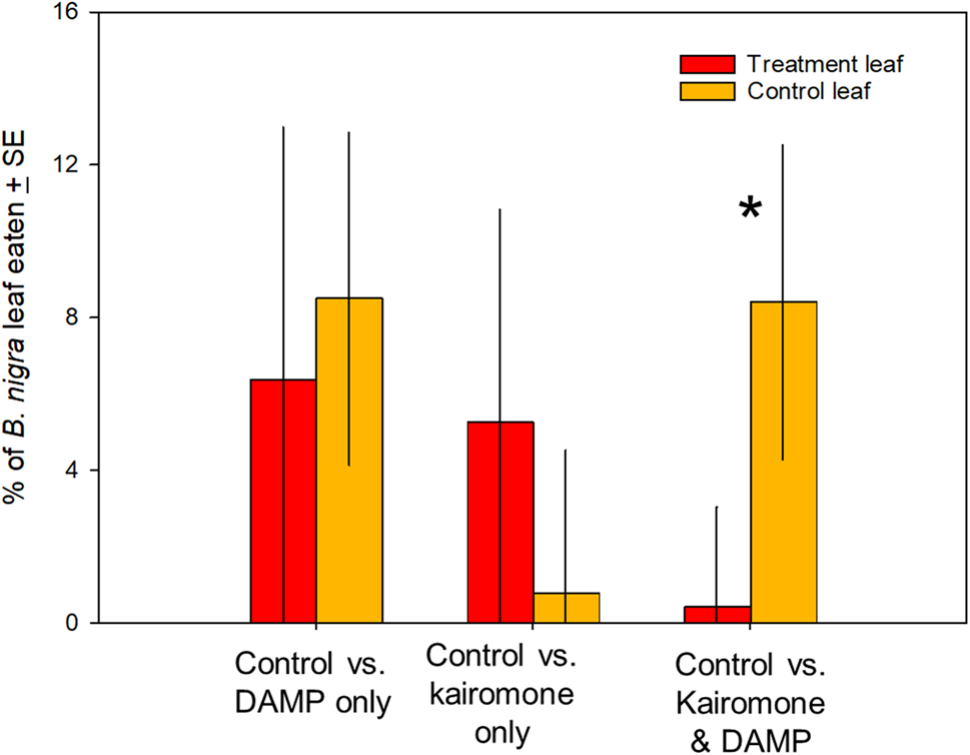
Proportion of plants eaten by *S. exigua* larvae when allowed to choose between leaves from the four treatments. **A** Leaves from control versus DAMP-only plants, **B** leaves from control versus kairomone-only, **C** leaves from control versus kairomone + DAMP plants. *S. exigua* did not differentiate between control plants and single treatment plants, but did prefer control plants over those treated with both pre-attack cues. ^*^p < 0.05

*Spodoptera* larvae did not significantly discriminate between control leaves and those exposed to kairomones or DAMPs separately (all *p* < 0.10). In contrast, *Spodoptera* did exhibit a preference when choosing between control leaves and those from plants exposed to both kairomones and DAMPs (Fig. 3). Larvae consumed 8.4% of controls versus > 1% of leaves from plants exposed to both risk cues (F_1,13_ = 6.47, *p* = 0.025).

## Discussion

Our results provide the first evidence that seeds and seedlings can integrate information from both herbivore kairomones and damage-associated molecular patterns (DAMPs) in their assessment of attack risk. Such induced responses to herbivory in plants provide essential defense against subsequent damage (Karban 2020). Since mature plants can generally wait until being attacked to induce defense (Sheriff et al. 2020), plant–herbivore research has focused on attack-associated cues such as tissue damage or volatile emissions. The ability to detect and respond to pre-attack cues like herbivore kairomones or DAMPs should be particularly beneficial for seeds and seedlings, life stages with limited resources for which herbivory is often lethal (Barton & Hanley 2013). While kairomone-induced increases in germination speed may seem counterintuitive, we hypothesize that earlier germination in the presence of risk cues from seed predators facilitates the resource acquisition necessary to mount effective defense (Pellegrini et al. 2024). Since defensive induction can be energetically costly, selection should also favor the use of multiple sources of pre-attack information to reduce undefended attacks while avoiding the cost of unnecessary defense (Orrock et al. 2015); our findings are consistent with this hypothesis.

For *B. nigra* seeds and seedlings, our results demonstrate that pre-attack cues indicating the proximity of both herbivores and damaged conspecifics altered both seedling germination and herbivore susceptibility (i.e., heighten a plant’s awareness of danger in the vicinity). This finding parallels research in predator–prey systems showing that cues from predators that consume prey conspecifics (akin to an herbivore whose presence produces kairomones and whose feeding produces DAMPs) are likely more dangerous than those that do not (Scherer & Smee 2016). Terrestrial and aquatic studies confirm that prey respond more to cues from conspecific-fed predators than to either predator (in our study, kairomone) or crushed conspecific (in our study, DAMP) cues alone (Prada et al. 2018; Schoeppner & Relyea 2005). Cues from predators fed conspecific or congeneric prey consistently elicit stronger responses than cues produced by the consumption of distantly related species (Schoeppner & Relyea 2009a; Schoeppner and Relyea 2009b; Shabani et al. 2008), arguing for the adaptive benefits of such gradated responses in both animal prey and plants.

While we predicted that exposure to combined risk cues would induce defense, we were surprised that neither cue induced defense by themselves. This was unexpected because both herbivore kairomones and DAMPS have been shown singly to induce pre-attack plant defense. Helms et al. (2013) reported that *Solidago* responds to the sex attractant of a gall-making fly, for instance, and Orrock (2013) found that snail mucus alters *B. nigra* growth and defense. We now know that cultivated and wild plants respond to slug and snail mucus (Falk et al. 2014; Kastner et al. 2014; Meldau et al. 2014; Orrock 2013; Orrock et al. 2018), and prior work found that *B. nigra* seeds and seedlings detect and respond to *A. subfuscus* (Pellegrini et al. 2024). Leaf homogenate from crushed conspecific plants similarly induces resistance in unwounded plants (Duran-Flores & Heil 2014); parallel responses to damaged-self cues occur in a range of taxa (Heil & Land 2014). Research demonstrating damaged-self responses to conspecific homogenates also found less induction in response to congeneric homogenates and none in response to crushed plants from different genera (Duran-Flores & Heil 2014). Although our work can be seen as contradictory to these findings, we suspect that aspects of our experimental design (detailed below) inadvertently reduced our ability to detect single-cue effects.

Faster germination in response to risk cues has only recently been documented (Pellegrini et al. 2024), but the impact of modest changes in germination on a plants’ life has been previously demonstrated (Orrock & Christopher 2010). We observed an increase in germination speed for seeds treated with both kairomone and DAMPs. This appears to be the first example of DAMPs acting as germination promoters in plants. Since risk cues changed germination speed but not the overall rate, detecting such shifts requires around-the-clock germination checks over a multi-day period; this may explain why our work is the first to identify this effect. While seedlings emerging from faster germinating seeds typically tend to outcompete their slower germinating counterparts (Orrock & Christopher 2010), we found no difference in seedling biomass; Pellegrini et al (2024) actually found that faster germinating seeds produced smaller seedlings. Both sets of results support the hypothesis that risk-induced changes in germination speed improve defense rather than competitive ability, an explanation consistent with the narrative that seeds must act quickly to avoid predation. While Pellegrini et al (2024) found that seeds with kairomone-induced increases in germination speed produced smaller seedlings, we observed no difference in final biomass (likely because we allowed our experiment to run long enough for the plants to all reach maturity). Our studies do, however, parallel others in linking faster germination to higher plant fitness (Karban 2008; Orrock & Christopher 2010).

There are several caveats to be considered in interpreting the results of our work. First, our finding that herbivore kairomones alone did not impact *B. nigra* seeds and seedlings contrasts with previously published research in this system (Pellegrini et al. 2024). The inability of *A. subfuscus* mucus alone to alter germination speed may result from our use of previously frozen mucus-covered soil rather than freshly collected material. While plant DAMPs do not appear to degrade after being frozen for a 16-week period (Duran-Flores & Heil 2014) and our pilot experiment found that the germination-accelerating effect of *A. subfuscus* slime persisted after nearly 5 months of freezing, freshly produced mucus from *B. nigra*-fed slugs may have contained DAMPs or some other volatile cues absent from the frozen soil we used. Second, both Pellegrini et al (2024) and Orrock (2013) harvested their *B. nigra* seedlings at 21 days, too small a size to conduct glucosinolate analyses on individual plants. Because of our interest in glucosinolates as a mechanism for herbivore defense, we allowed plants in our experiment to grow to maturity (36 days) before harvesting them. While this ensured we had sufficient aboveground biomass for chemical analyses, it may also have allowed kairomone-and-DAMP-treated seedlings that started out smaller the time to ‘catch up’ in size to control seedlings. This difference in size may also explain both the absence of among-treatment differences in glucosinolate levels and the minimal herbivory in all treatments. *Spodoptera* consumed 5 ± 1.5% [SE] of leaf biomass in our work, while *A. subfuscus* herbivory in Pellegrini et al (2024) averaged 31 ± 11.2%. In addition to being larger, leaves from older plants also possessed trichomes and were significantly tougher to tear; while we know in most cases that seedlings are generally more palatable to mollusks than adults (Fenner et al. 1999), future research should address how physical defenses or non-glucosinolate chemical defenses might affect herbivore consumption. Additional work should explore the effect of individual and combined cues on the growth, chemical defense, and palatability of cotyledons and smaller true leaves, since both are produced when treated plants are likely more focused on defense.

The fact that plants utilized multiple risk cues to alter defensive investment provides avenues for future research. For example, a lack of impact of crushed plant alone on time to radicle emergence may warrant further lab studies with isolated molecular patterns found within certain DAMPs (Tanaka & Heil 2021). In addition, *B. nigra* and *A. subfuscus* co-occur in their native range; the substantial impact of *Arion* on endemic North American plants may reflect the lack of a coevolutionary history that would otherwise allow them to employ risk-induced defense. Interplay between risk cues might also alter interactions with plants and other non-molluscan herbivores (Orrock et al. 2018) or affect competition between conspecific or heterospecific seedlings that differ in their vulnerability to attack risk (Hanley et al. 1995; Orians et al. 2013). Since herbivory in the parental generation can increase defense in their offspring (Agrawal et al. 1999), it would also be of interest to see whether kairomone and DAMP exposure in the absence of actual herbivory would alter the cue receptivity or response in their offspring.

## Supplementary Information

Below is the link to the electronic supplementary material.

Supplementary file1 (DOCX 108 KB)

## Data Availability

Should the paper be provisionally accepted, the authors pledge to archive all data on figshare prior to final acceptance. This will be the permanent repository.
