# Correction: Herbivore cues and plant damage-associated compounds jointly alter seed germination and seedling herbivory

**DOI:** 10.1007/s00442-025-05859-1

**Published:** 2026-02-05

**Authors:** Katherine M. Overstrum, Eirette M. Santiago, Brooke A. Pellegrini, Kevin C. Headrick, Colin M. Orians, John L. Orrock, Evan L. Preisser

**Affiliations:** 1 Department of Biological Sciences, University of Rhode Island, Kingston, RI 02881 USA; 2 Department of Biology, Tufts University, Medford, MA USA; 3 Department of Integrative Biology, University of Wisconsin, Madison, WI USA

**Correction: Oecologia (2025) 208:1** 10.1007/s00442-025-05831-z

In this article Fig(s) 1, 2 and 3 appeared incorrectly and have now been corrected in the original publication. For completeness and transparency, the old incorrect versions are displayed below.

Incorrect version:


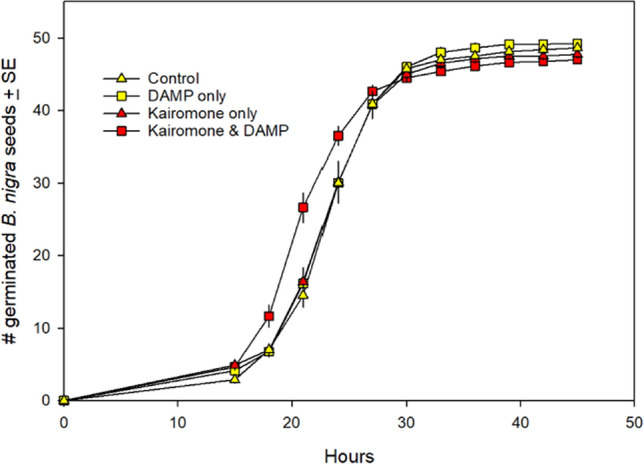


**Fig. 1** Effect of exposing *B. nigra* seeds to control (soil and water only), kairomone only, DAMP only, or both kairomone and DAMP prior to and during germination. Seeds exposed to both kairomone and DAMP germinated significantly faster


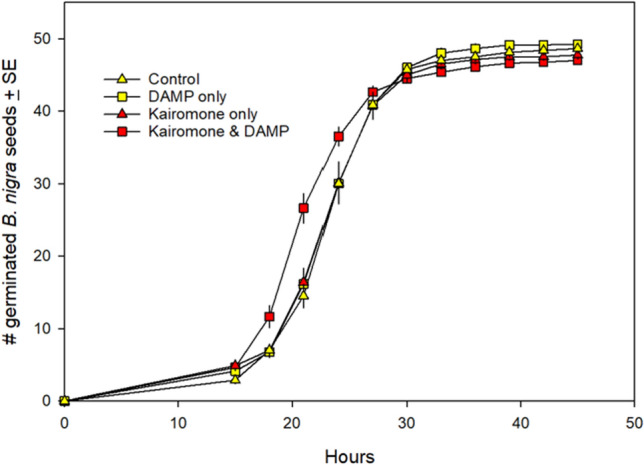


**Fig. 2** Effect of exposing *B. nigra* seeds and seedlings to control (soil and water only), kairomone only, DAMP only, or both kairomone and DAMP. There was no significant difference in aboveground, below-ground, or total biomass for seedlings at the time of harvest


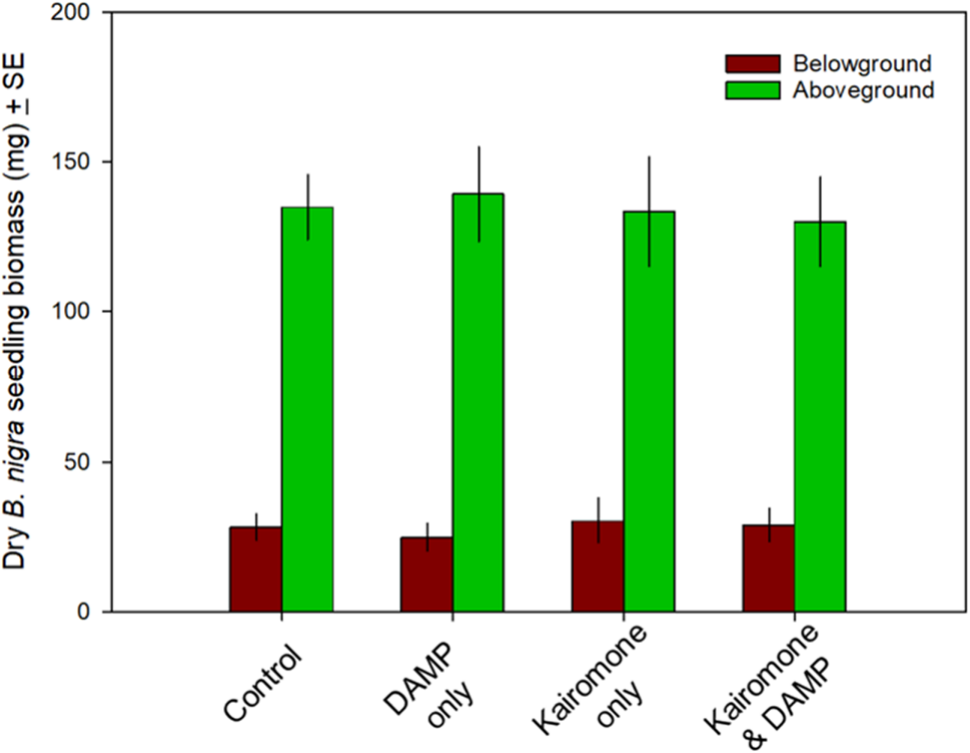


**Fig. 3** Proportion of plants eaten by *S. exigua* larvae when allowed to choose between leaves from the four treatments. **A** Leaves from control versus DAMP-only plants, **B** leaves from control versus kairomone-only, **C** leaves from control versus kairomone + DAMP plants. *S. exigua* did not differentiate between control plants and single treatment plants, but did prefer control plants over those treated with both pre-attack cues. *p < 0.05

Corrected version:


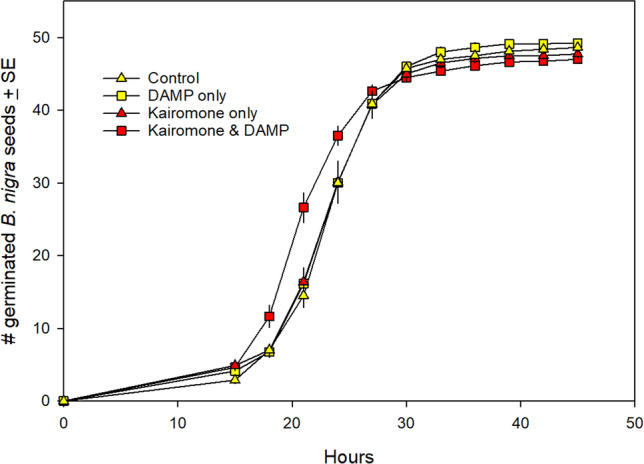


**Fig. 1** Effect of exposing *B. nigra* seeds to control (soil and water only), kairomone only, DAMP only, or both kairomone and DAMP prior to and during germination. Seeds exposed to both kairomone and DAMP germinated significantly faster


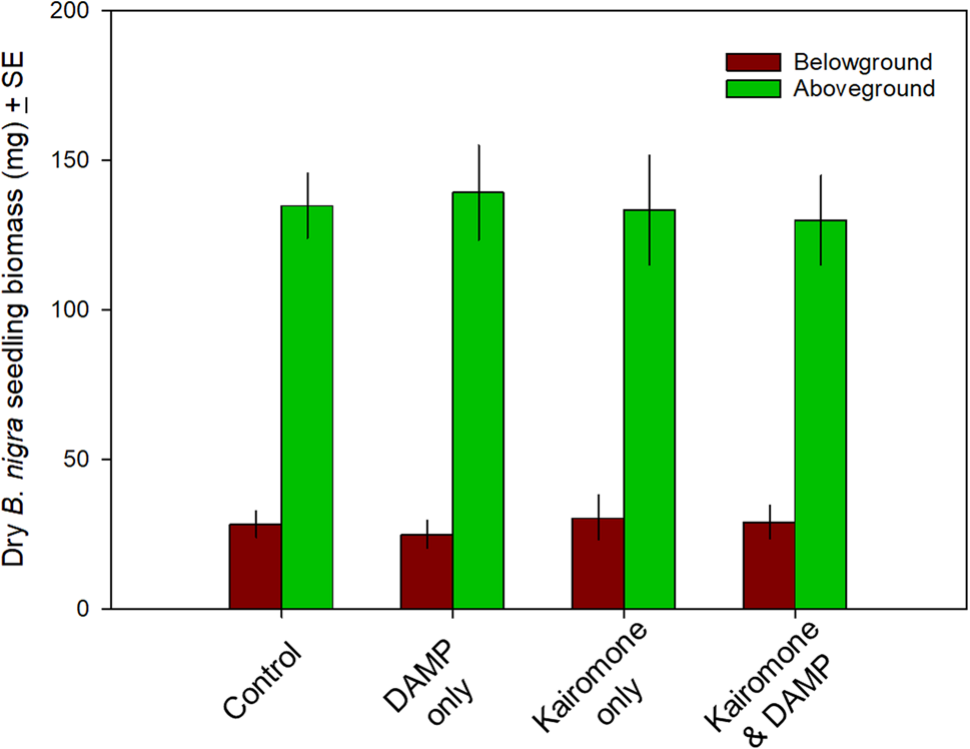


**Fig. 2** Effect of exposing *B. nigra* seeds and seedlings to control (soil and water only), kairomone only, DAMP only, or both kairomone and DAMP. There was no significant difference in aboveground, below-ground, or total biomass for seedlings at the time of harvest


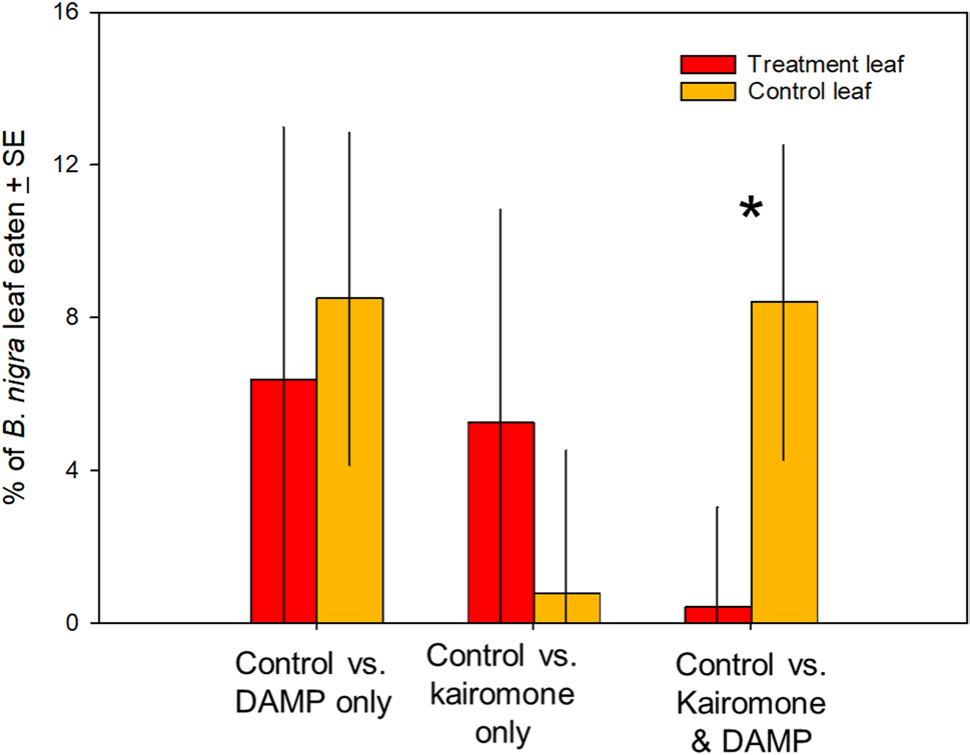


**Fig. 3** Proportion of plants eaten by *S. exigua* larvae when allowed to choose between leaves from the four treatments. **A** Leaves from control versus DAMP-only plants, **B** leaves from control versus kairomone-only, **C** leaves from control versus kairomone + DAMP plants. *S. exigua* did not differentiate between control plants and single treatment plants, but did prefer control plants over those treated with both pre-attack cues. *p < 0.05

The original article has been corrected.

