# Supplementary material for: Herbivore cues and plant damage-associated compounds jointly alter seed germination and seedling herbivory
Source: Oecologia. 2025 Nov 25;208(1):1. doi: 10.1007/s00442-025-05831-z (PMC12647282; doi:10.1007/s00442-025-05831-z)

**Supplementary appendix #1: Freezing slime experiment**

**Methods:** *Brassica nigra seeds* were sourced from Outsidepride Seed Source, LLC, Independence, OR USA). *Arion subfuscus* was obtained from URI’s Kingston Wildlife Research Station and maintained in 10 terrariums in the lab. They were misted and fed three times weekly on a diet of organic romaine lettuce, organic carrots, and hemp hearts.

One day before the start of the experiment we added soil (5.0 g of sifted MiracleGro Potting Mix moistened with distilled water in a 1:4 ratio) to each of 100 60-mm petri plates. We then added a single mature *A. subfuscus* (~0.5 g) to each of 50 plates (frozen-cue treatment); the other 50 plates did not receive any slugs (control treatment). The treatment and control plates were then interspersed and held in a dark cabinet in the laboratory at 20°C. After one day, plates were retrieved from the cabinet and slugs removed from each of the treatment plates. All 100 plates were then stored in a -18°C freezer from 29 September 2023 until their use.

*November 2023 assay (39 days post-freezing):* On 6 November 2023 we followed the above procedure to generate ten 60-mm ‘fresh cue’ treatment plates. Briefly, ten plates were prepared as above and a single *A. subfuscus* added to each at 12:00 hours. At 10:00 hours on 7 November 2023, ten control and ten treatment plates were removed from the freezer and allowed to defrost. Two hours later, slugs were removed from each of the ten fresh-cue plates and the experiment began. The soil in each of the 30 petri plates (ten frozen control, ten frozen cue, ten fresh cue) was transferred to a new 60 mm petri plate lined with a 60mm white filter paper disk moistened with 1 ml of distilled water. After the soil was transferred, a 60 mm black filter paper disk was placed on top of the soil and moistened with 1 ml of distilled water; black filter paper was used in this step because a white radicle is more easily detected against a dark background. Twenty *B. nigra* seeds were then placed on top of the black filter paper in each of the 30 petri plates. Plates from the different treatments were then interspersed on a tray and held in a Percival 6 growth chamber in total darkness at 21°C. The number of germinating seeds per plate was recorded starting at 20 hours and every three hours thereafter until hour 53 (when each plate contained at least 10 germinating seeds).

*December 2023 assay (74 days post-freezing):* The assay started on 12 December 2023 and was identical in design to the November 2023 experiment except that it did not include a fresh-cue treatment (all adult *A. subfuscus* had reproduced and died). During the incubation period, the temperature gauge in the growth chamber malfunctioned and temperatures spiked to 25°C. As a result, we did a germination check at hour 18, again at hour 20, and then every three hours until hour 38 (when each plate contained at least 10 germinating seeds).

*March 2024 assay (157 days post-freezing):* The assay started on 4 March 2024 and was identical in design to the December 2023 experiment and involved 30 replicates (15 control, 15 frozen cue). Germination checks started at hour 23 and every three hours thereafter until hour 59.

**Analysis:** Data for all three experiments was analyzed using rm-ANOVA; we report univariate unadjusted epsilon values for the within-subject terms ‘time’ and ‘time*treatment’. All analyses were conducted using JMP 18.1.2.

**Results and Figures**

**Figure 1: Fresh *A. subfuscus*** **mucus vs mucus frozen for 40 days vs. frozen control**

Both fresh and frozen *A. subfuscus* mucus accelerated germination relative to the frozen control (treatment F_2,27_ = 5.09, p = 0.013; time F_11,297_ = 444, p < 0.001; time*treatment F_22,297_ = 3.27, p < 0.001).


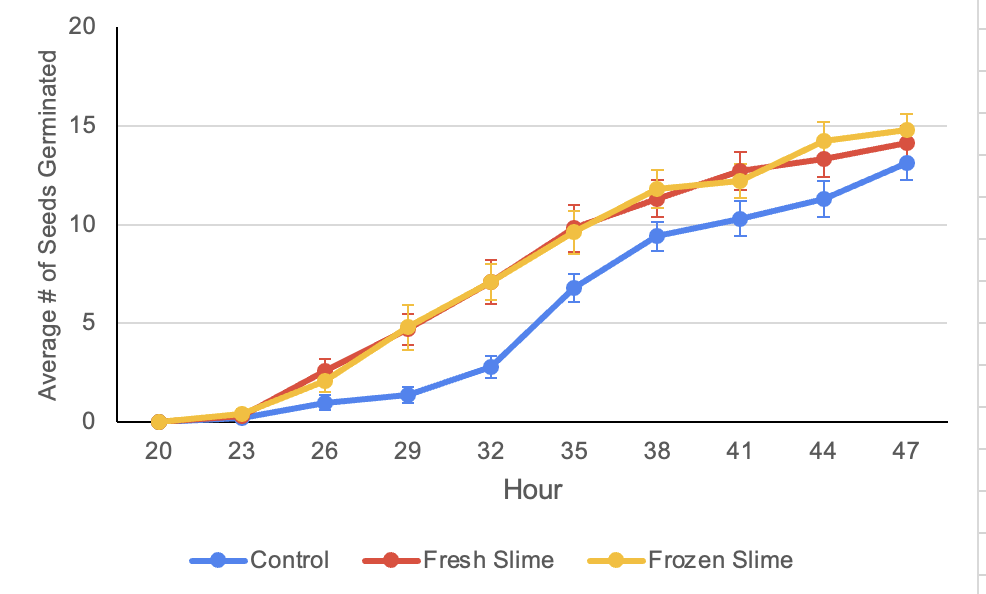


**Figure 2: Mucus frozen for 75 days vs. frozen control**

Although there was no main effect of treatment (F_1,18_ = 1.63, p = 0.22), there was a significant time and time*treatment interaction (time F_7,126_=329, p < 0.001; time*treatment F_7,126_ = 2.11, p = 0.047).


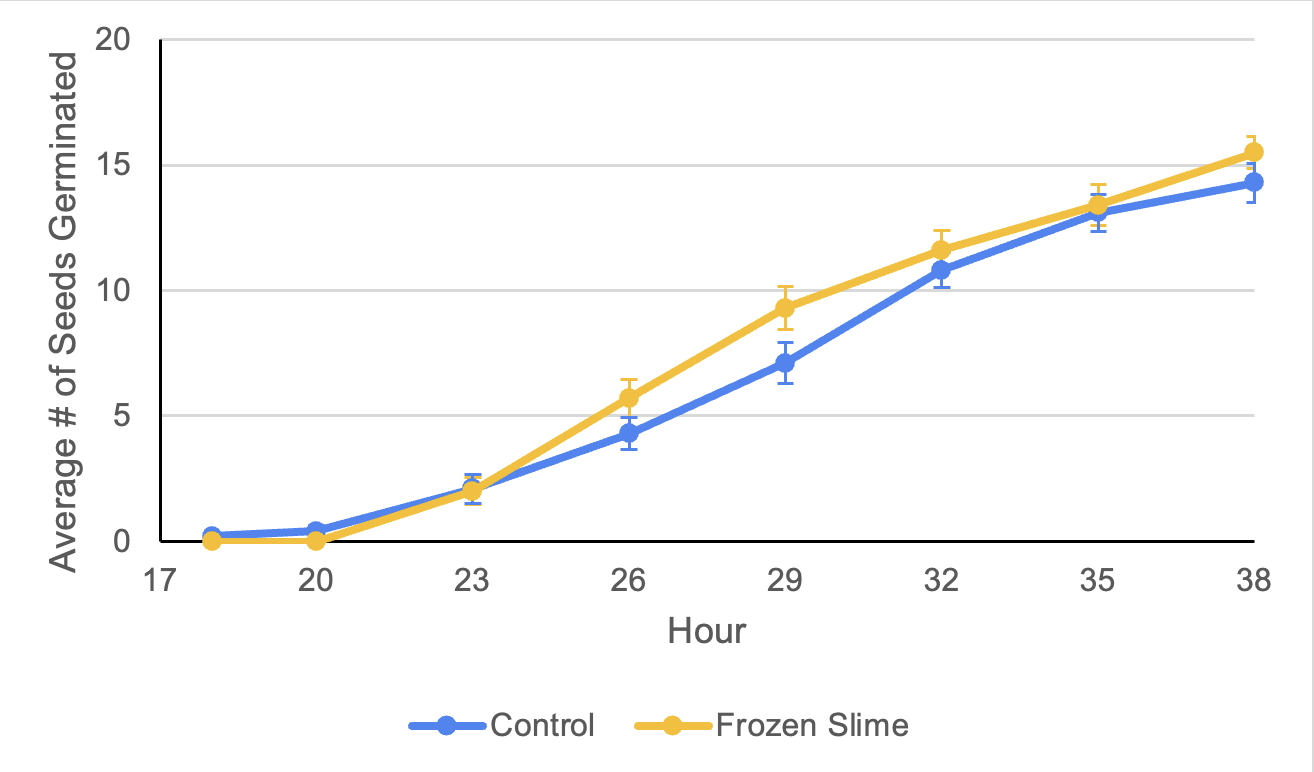


**Figure 3: Mucus frozen for 140 days vs. frozen control**

Frozen *A. subfuscus* mucus accelerated the number of seeds germinating and germination time relative to the frozen control (treatment F_1,28_ = 4.76, p = 0.038; time F_12,336_ = 516, p < 0.001; time*treatment F_12,336_ = 4.05, p < 0.001).


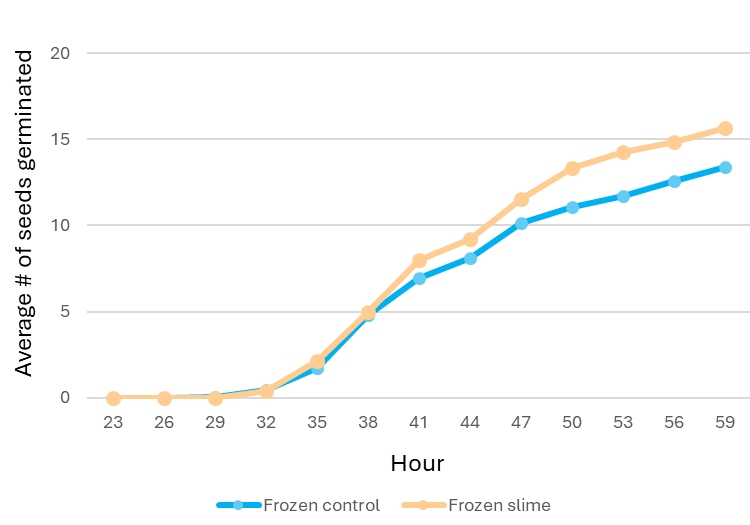

Supplement: Supplementary file 1 — Supplementary file1 (DOCX 108 KB) [file 442_2025_5831_MOESM1_ESM.docx]
